# Résultats de la chirurgie laparoscopique pour la hernie de l’aine: l’expérience Tunisienne

**DOI:** 10.11604/pamj.2018.29.43.14013

**Published:** 2018-01-17

**Authors:** Houcine Maghrebi, Amin Makni, Amin Sebai, Faouzi Chebbi, Wael Rebai, Amin Daghfous, Rachid Ksantini, Mohamed Jouini, Montassar Kacem, Zoubeir Ben Safta

**Affiliations:** 1 Département de Chirurgie «A», La Rabta Hôpital, Faculté de Médecine de Tunis, Université de Tunis, El Manar, Tunisie

**Keywords:** Hernie inguinale, chirurgie, plaque, laparoscopie, Inguinal hernia, surgery, plate, laparoscopy

## Abstract

La hernie de l'aine de l'adulte reste une affection fréquente en chirurgie digestive. De nombreuses techniques de réparation ont été décrites à ce jour dont les procédés laparoscopiques. Deux méthodes furent rapidement adoptées par les différents praticiens pour le traitement chirurgical des hernies de l'aine par laparoscopie: la méthode laparoscopique totalement extra péritonéale (TEP) et la méthode laparoscopique transpéritonéale (TAPP). Le but était d'étudier la faisabilité de la cure de hernie de l'aine par cœlioscopie et de décrire ses résultats du point de vue récidive herniaire et douleur post opératoire. Ce travail était une étude rétrospective, uni centrique, et transversale, portant sur des patients opérés par des chirurgiens du service de chirurgie A La Rabta pour hernie de l'aine par voie laparoscopique, sur une période de 8 ans allant de janvier 2006 à décembre 2013. Le principal critère de jugement était la récidive herniaire. La douleur post opératoire et les complications étaient les critères de jugement secondaires. Nous avons colligés 104 hernies chez 92 patients respectant les critères d'inclusion de notre étude. La moyenne d'âge de nos patients était de 48 Ans (19-83). L'approche TAPP était la plus utilisée: 94 cas (90%) TAPP contre 10 cas TEP. Aucune complication per opératoires n'a été signalée. Le taux de conversion de notre série était nul. La mortalité opératoire était aussi nulle. La morbidité postopératoire était de 5% (5 patients). Elle était à type d'hématome dans 3 cas et de sérum dans 2 cas. La durée moyenne d'hospitalisation était de 1.2 jours (1-4jours). Le séjour post opératoire n'avait pas dépassé 2 jours chez 94% des patients. Seulement 2 patients avaient présenté une récidive. Les douleurs chroniques postopératoires étaient notées chez seulement 3 patients. Notre étude a montré que la cure de hernie de l'aine par laparoscopie a apporté un confort considérable à nos patients en ce qui concerne les phénomènes douloureux, les durées d'hospitalisation et d'arrêt de travail. Les résultats obtenus dans cette série sont bons et conformes aux résultats déjà publiés dans la littérature. Ceci nous encourage à poursuivre l'utilisation de ces techniques et à contrôler nos résultats à plus long terme.

## Abstract (en)

Groin hernia in adults is a frequent affection in digestive surgery. Many repair techniques have been described to date, including laparoscopic surgery. Two methods are quickly adopted by the various practitioners for the surgical treatment of groin hernia using laparoscopy: laparoscopic totally extra-peritoneal (TEP) technique and laparoscopic transperitoneal inguinal hernia repair (TAPP). This study focused on the feasibility of groin hernia repair using coelioscopy, aiming to describe its outcomes in terms of recurrence and postoperative pain. We conducted a single-center, retrospective and cross-sectional study of patients who had undergone laparoscopic surgery for groin hernia repair in the Department of Surgery at La Rabta Hospital over a period of 8 years, from January 2006 to December 2013. The main evaluation criterion was hernia recurrence. Postoperative pain and complications were the secondary criteria of judgment. We collected data from 92 patients with 104 hernias, respecting the inclusion criteria in our study. The average age of patients was 48 years (19-83). TAPP was the most used technique: 94 TAPP (90%) versus 10 TEP. No intraoperative complication was reported. Conversion rate was 0. Operative mortality was also 0. Postoperative morbidity was 5% (5 patients). It included hematoma in 3 cases and serum in 2 cases. The mean lenght of stay in hospital was 1.2 days (1-4 days). Postoperative length of stay didn’t exceed 2 days in 94% of patients. Only 2 patients had a recurrence. Postoperative chronic pain was reported only in 3 patients. Our study shows that laparoscopic surgery for groin hernia repair gives considerable comfort to our patients with regard to pain, length of stay in hospital and cessation of work. Outcomes are good and consistent with the results already published in the literature. This encourages surgeons to use these techniques and to monitor longer term outcomes.

## Introduction

La cure des hernies de l'aine occupe une place importante dans l'activité d'un service de chirurgie générale. Elle constitue la deuxième intervention la plus fréquente, venant au 2^ème^ rang après les appendicectomies. On estime que 20 millions de hernies inguinales sont opérées dans le monde chaque année [1]. Plusieurs techniques de réparations ont été décrites. Deux méthodes sont actuellement utilisées: la méthode laparoscopique totalement extrapéritonéale (TEP) et la méthode laparoscopique transpéritonéale (TAPP). La cure laparoscopique des hernies de l'aine a des avantages démontrés par rapport aux voies conventionnelles: préjudice esthétique moindre, moins de douleur post opératoire, reprise plus rapide d'activité physique, professionnelle et sportive. Le but était d'étudier la faisabilité de la cure de hernie de l'aine par cœlioscopie et de décrire ses résultats du point de vue récidive herniaire.

## Méthodes

Il s'agit d'une étude rétrospective, uni centrique, et transversale, portant sur 92 patients opérés par des chirurgiens du service de chirurgie A La Rabta pour hernie de l'aine par voie laparoscopique. Ces patients ont été colligés durant une période de 8 ans allant de Janvier 2006 à Décembre 2013.Cette étude a inclus tous les malades âgés de 18 ans et plus sans limite supérieure d'âge présentant des hernies de l'aine non compliquées. Nous avons exclu les patients présentant des contre-indications à la cœlioscopie ou à l'anesthésie générale. Le critère principal de jugement principal était le taux de récidive. Les critères secondaires étaient la morbidité per et postopératoire et les douleurs chroniques post opératoires. Tous les malades ont été opérés par des chirurgiens séniors ayant une expertise en chirurgie laparoscopique. Les techniques de réparation étant standardisées dans notre service.

**Figure 1:**
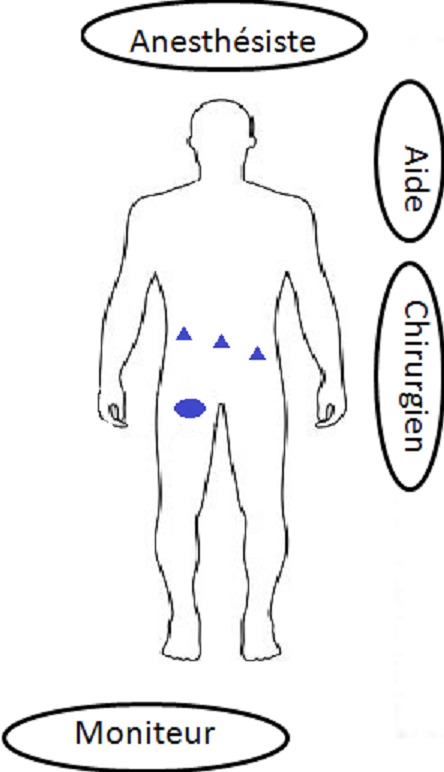
Installation du patient et disposition des trocarts

**Figure 2:**
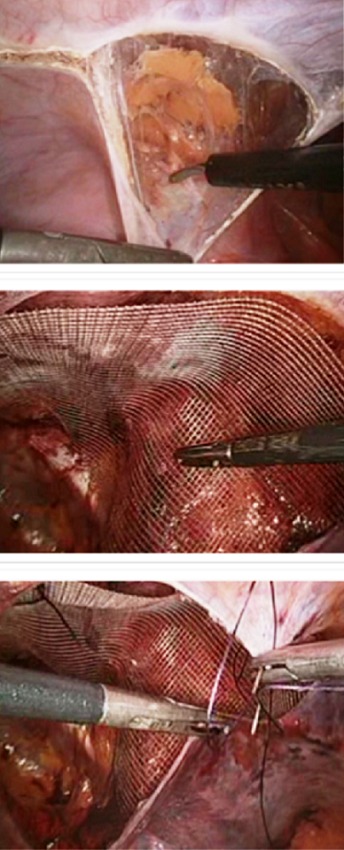
Positionnement de la plaque et fermeture du péritoine

**Technique de la TAPP:** Le patient est installé en décubitus dorsal les deux bras le long du corps, membres inférieurs joints, avec un léger Trendelenburg. L'opérateur se place du coté opposé à la hernie. La colonne de c'lioscopie sera placée aux pieds du malade. Trois trocarts sont utilisés: Un trocart de 10 mm au bord supérieur de l'ombilic pour l'optique et pour l'introduction ultérieure de la prothèse. Deux trocarts de 5 mm au niveau des flancs droit et gauche, à distances équivalentes, sur une ligne droite qui passe par l'ombilic et qu'on oriente vers le coté de la hernie (Figure 1). La cure de la hernie passe par 4 étapes primordiales: Ouverture de l'espace pré péritonéal, dissection du sac herniaire, introduction et positionnement de la prothèse et enfin la péritonisation (Figure 2).

**Technique de la TEPP:** Après création du pneumo-Retzius, on procède à la mise en place des trocarts, dissection de l'espace de Bogros, dissection de la hernie et enfin l'introduction et le positionnement de la prothèse. Au cours de ce travail, l'analyse statistique a été réalisée au moyen du logiciel SPSS version 17.0. Nous avons calculé des fréquences absolues et des fréquences relatives (pourcentages) pour les variables qualitatives. Nous avons calculé des moyennes, des médianes et des écarts-types et déterminé les valeurs extrêmes pour les variables quantitatives. Dans tous les tests statistiques, le seuil de signification a été fixé à 0,05.

## Résultats

Nous avons colligés 104 hernies chez 92 patients respectant les critères d'inclusion de notre étude. La moyenne d'âge de nos patients était de 48 ans avec des extrêmes allant de 19 à 83 ans. Une nette prédominance masculine a été notée. Soixante-dix de nos patients avaient un emploi avant l'intervention soit 76%, dont 16 patients faisaient un travail de force. Cinq patients pratiquaient une activité sportive de façon régulière; ce qui représente 5% des malades. Sur le plan chirurgical, deux techniques laparoscopiques ont été utilisées: la TAPP et la TEP. L'approche TAPP était la plus utilisée: 94 cas (90%) TAPP contre 10 cas TEP. Le matériel prothétique utilisé était du polypropylène dans 9 cas, du mersylène dans 4 cas, une plaque à grippe chez 10 patients et une prothèse 3D dans 19 cas. Le type de prothèse utilisé n'a pas été précisé dans les autres cas. Vingt sept plaques ont été fixées au fil. 37 l'ont été aux agrafes non résorbables. La prothèse n'a pas était fixées dans 30 cas. Aucune complication peropératoire n'a été signalée et aucune conversion n'a été répertoriée. Un drainage a été réalisé dans 6 cas, enlevé dans tous les cas au premier jour postopératoire. Soixante interventions (64%) n'avaient pas dépassé les 60 minutes. La mortalité opératoire de notre série était nulle. La durée moyenne d'hospitalisation était de 1,2 jour (1-4jours). Quatre vingt quatorze pourcent des patients n'avaient pas dépassé 2 jours de séjour post opératoire. La morbidité postopératoire était de 5% (5 patients). Elle était à type d'hématome dans 3 cas et de sérum dans 2 cas. Aucune complication médicale n'a été signalée. Le délai de reprise de l'activité normale était de 21 jours. Le recul moyen de notre série était de 2 ans. Huit patients étaient perdus de vue après la première consultation de contrôle. Seulement 2 patients avaient présenté une récidive: un patient opéré par TAPP et qui avait récidivé au bout de 7 mois (réopéré par voie inguinale) et un deuxième patient opéré par TEP qui avait présenté une récidive au bout de 10 mois (réopéré par TAPP). Par ailleurs, des douleurs chroniques postopératoires ont été notées chez trois patients: deux d'entre eux étaient opérés selon la technique TAPP (fixation de la plaque réalisée par les agrafes non résorbables) et un patient opéré par la technique TEP.

## Discussion

Notre étude a démontré que la réparation par abord laparoscopique offre des avantages incontestables de point de vu durée opératoire, résultat esthétique, complications pariétales, confort post opératoire et retour à l'activité professionnelle. Pour ce qui est des complications per-opératoires, elles sont nulles dans notre série. Dans la littérature, le taux des complications per-opératoires [1-4] varie de 1,3 à 36% [5,6]. Ceci s'explique par l'expertise de notre équipe en matière de chirurgie laparoscopique. Quant aux complications postopératoires, elles étaient de 5% (5 patients). Une analyse de la littérature a montré un taux global de complications post opératoires qui varie de 3 à 39% [7]. Plusieurs études montrent plus de complications après une chirurgie ouverte qu'en cœlioscopie, d'autres séries montrent plus de complications après la voie laparoscopique qu'en chirurgie classique [1]. La prévention de ces complications repose en premier lieu sur une dissection minutieuse du sac herniaire, le respect et une bonne connaissance des composants du cordon spermatique et des vaisseaux de la région.

La durée du séjour post opératoire pour les patients ayant bénéficié d'un procède laparoscopique dépasse rarement 2 ou 3 jours [8-16]. Dans notre série, la durée moyenne d'hospitalisation était de 1.2 jours (1 à 4 jours). Ceci s'explique essentiellement par la diminution de la douleur post opératoire et le concept de réhabilitation précoce. La chirurgie herniaire laparoscopique a également permis de diminuer la douleur post opératoire permettant ainsi le retour rapide à l'activité professionnelle. Le délai de reprise de l'activité normale était de 21 jours dans notre série en concordance avec les données de la littérature. Les douleurs chroniques à distance restent le point fort de ces techniques. Elles permettent de diminuer le taux des névralgies et des paresthésies, ce qui est à l'origine d'un confort et un bien être plus important chez les opérés. Ces douleurs chroniques postopératoires ont été notées chez seulement 3 patients. Les taux rapportés dans la littérature sont de 0 à 23% [17-22]. Enfin, concernant la récidive qui est le paramètre essentiel à prendre en considération dans l'adoption de chaque technique, le taux de récidive (1,9%) était concordant avec les données de la littérature [21-24] (Tableau 1).

**Tableau 1:** Taux de récidive dans la littérature

Auteurs	Technique	Taux de récidive
G. Tzovaras[6]	TAPP 94	4 (4,3%)
D. Birk [20]	TAPP 169	3 (1,77%)
Hidetoshi Wada [8]	TAPP 352	10 (2,84%)
Pavol Klobusicky [23]	TAPP 95	0 %
Florian Muschalla [24]	TAPP 1010	4 (0,4%)
Hiroki Toma [9]	TEP 303	4 (1,32%)
Dedemadi [22]	TAPP 24 TEP 25	2(8%) 2(8%)
Pokorny [10]	TAPP 84 TEP 35	4(5%) 2(6%)
Hamza [22]	TAPP 25 TEP 25	1(4%) 1(4%)
Gong [11]	TAPP 50	0%
	TEP 52	
Krishna [12]	TAPP 56	0%
	TEP 66	
Netto [22]	Ouverte 126	2(1,6%)
	TAPP 94 TEP24	2(1,7%)
Notre série	TAPP 94	2(1,9%)
	TEP 10	

Dans les dernières mises à jour des recommandations de l'International EndoherniaSociety, une comparaison entre TAPP et TEP a été réalisée [25]. Les auteurs avaient réalisé une revue systématique de la littérature dans les bases de données Cochrane database, PubMed database, Medline database. Ils avaient utilisé les mots clés: TAPP, TEP, TAPP versus TEP, Total Extra péritonéal repair, Trans abdominal Preperitoneal repair, Inguinal hernia. Un total de 200 publications ont été identifiées dont 11 étaient utilisées. Ils concluent avec de bons niveaux de preuve que les complications majeures sont rares pour les deux procédés. La TAPP avait par ailleurs une durée opératoire plus longue par rapport à la TEP. Cette dernière donnait plus de complications chirurgicales par rapport à la TAPP. L'*International Endohernia Society* [25] conclue ainsi avec un grade de recommandation A que les deux techniques représentent des options thérapeutiques acceptables et efficaces pour le traitement des hernies inguinales. Les données de la littérature sont insuffisantes pour conclure à la supériorité d'une technique par rapport à l'autre.

## Conclusion

La hernie de l'aine de l'adulte reste une affection fréquente en chirurgie digestive. De nombreuses techniques de réparation ont été décrites à ce jour, allant des herniorraphies passant par les cures prothétiques et arrivant aux techniques de réparation herniaire laparoscopique. Notre étude a montré les bénéfices de ces techniques laparoscopiques en diminuant les douleurs postopératoires immédiates ainsi que les douleurs chroniques, sans pour autant altérer les résultats à long terme. Concernant la récidive qui reste le paramètre essentiel à prendre en considération dans l'adoption de chaque technique, la comparaison entre les procédés classiques et la cœliochirurgie est difficile à réaliser en raison de la grande variabilité des techniques ouvertes. En prenant en compte cette réserve, le taux de récidive pour les deux techniques reste superposable.

### Etat des connaissances actuelle sur le sujet

- La hernie de l'aine de l'adulte reste une affection fréquente en chirurgie digestive;
- Elle constitue la deuxième intervention la plus fréquente;
- La cure des hernies de l'aine occupe une place importante dans l'activité d'un service de chirurgie générale.

### Contribution de notre étude à la connaissance

- Deux principales techniques de réparations cœlioscopies ont été adoptées par les différents praticiens;
- La cure de hernie de l'aine par laparoscopie offre un confort considérable aux patients en ce qui concerne les phénomènes douloureux, les durées d'hospitalisation et d'arrêt de travail;
- La comparaison entre les procédés classiques et la cœlio-chirurgie est difficile à réaliser en raison de la grande variabilité des techniques ouvertes.

## Conflits d’intérêts

Les auteurs ne déclarent aucun conflit d'intérêts.

## Remerciements

Nous remercions Pr Zouari Béchir pour son aide précieuse dans la partie statistique.
